# Supplementary material for: Genome-Wide DNA Methylation Profile Indicates Potential Epigenetic Regulation of Aging in the Rhesus Macaque Thymus
Source: Int J Mol Sci. 2022 Nov 29;23(23):14984. doi: 10.3390/ijms232314984 (PMC9738698; doi:10.3390/ijms232314984)
Supplement: Supplementary file 1 [file ijms-23-14984-s001.zip › Supplementary figures 1-8.ijms-2013450.pdf]

Supplementary Figures

Genome-wide DNA Methylation Profile Indicates Potential Epigenetic Regulation of Aging in the Rhesus Macaque Thymus

Authors: Hong Qiu , Haobo Li , Ruiwen Fan , Yang Song , Xuan Pan , Chunhui Zhang and Jing Li *

Description of Supplementary Figures

Supplementary Figure S1: Distribution of methylation levels and TPM of genes per each sample.

Supplementary Figure S2: Distribution of three contexts cytosine in the whole genome.

Supplementary Figure S3: Methylation level on CHH, CHG, CpG contexts cytosine in different genomic annotations across the genome.

Supplementary Figure S4: Distribution of different methylation regions (DMRs) on chromosomes.

Supplementary Figure S5: GO and KEGG pathway enrichment analysis of DMGs.

Supplementary Figure S6: Statistics of shared SNV, shared ASM sites, cASM and aDMRs.

Supplementary Figure S7: GO terms and KEGG pathways enrichment analysis of DEGs.

Supplementary Figure S8: Relationship between methylation and expression.

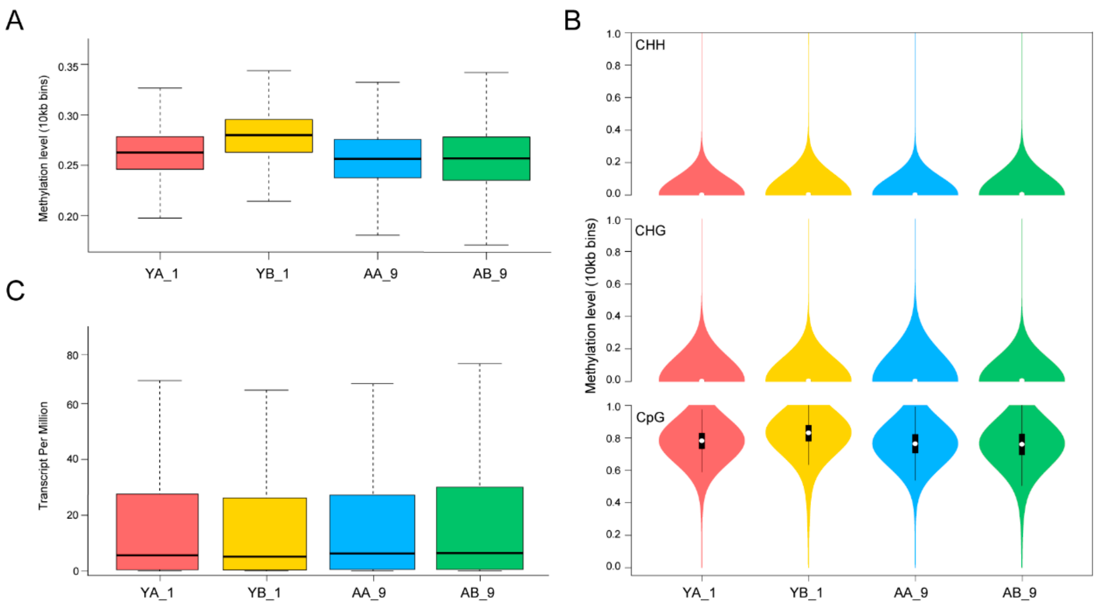

Figure S1. Distribution of methylation levels and TPM of genes per each sample. (**A**) Box plot indicates methylation expression with filtering outliers. Every 10kb was taken as a bin in box plot. (**B**) Violin plot indicates distribution of methylation levels on CHH, CHG, CpG contexts cytosine. The x-axis represents different samples and the y-axis represents methylation level. Every 10kb was taken as a bin in violin plot. The width of each violin indicates the number of points at that methylation level (**C**) Box plot indicates mRNA expression with filtering outliers.

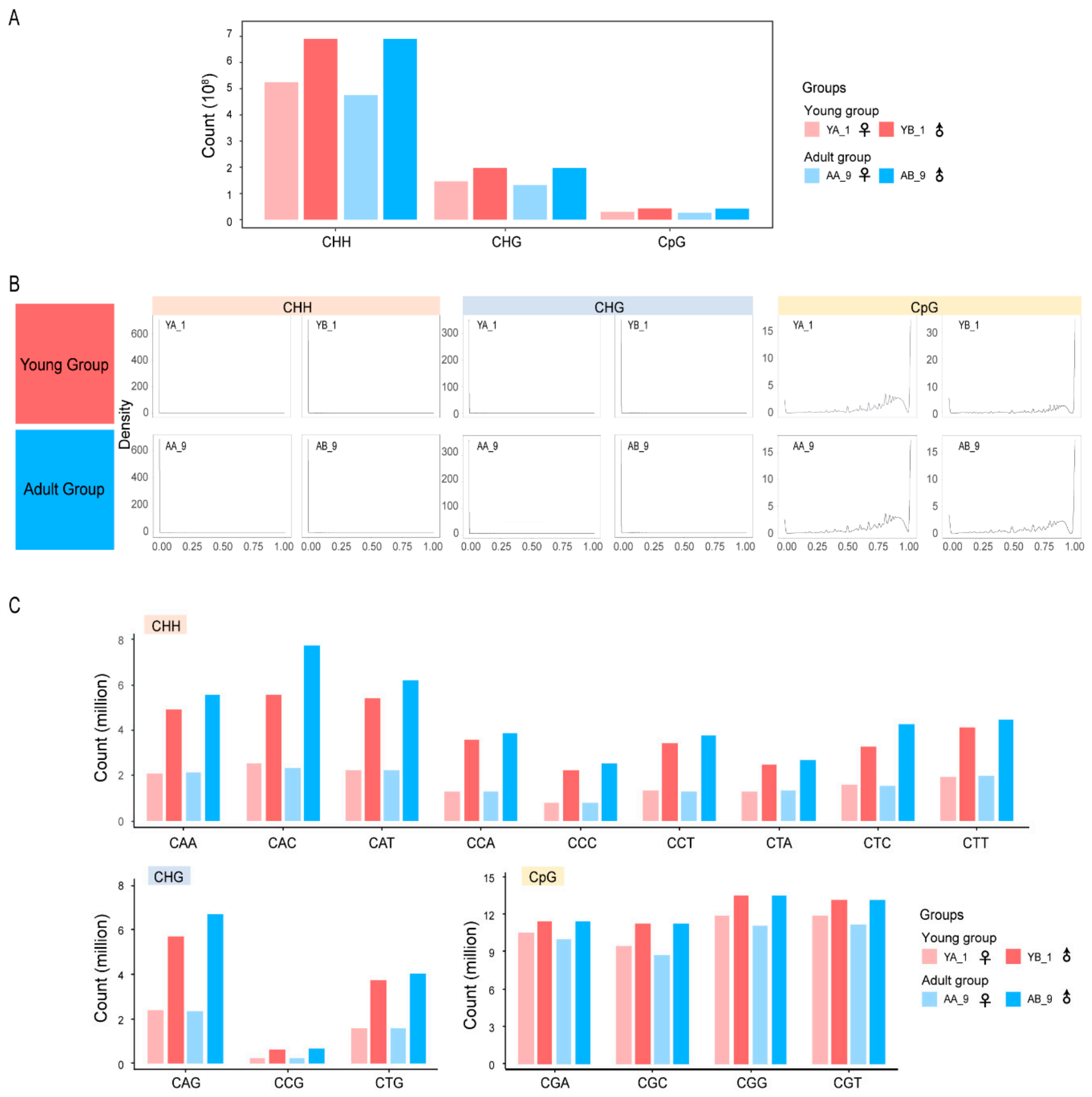

Figure S2. Distribution of three contexts cytosine in the whole genome. (**A**) Count of CHH, CHG and CpG contexts cytosine with more than 5 reads per cytosine in four individuals. (**B**) Density of the methylation level on CHH, CHG and CpG contexts cytosine with more than 5 reads per cytosine in four individuals. (**C**) Count of CHH, CHG and CpG contexts cytosine by trinucleotide context.

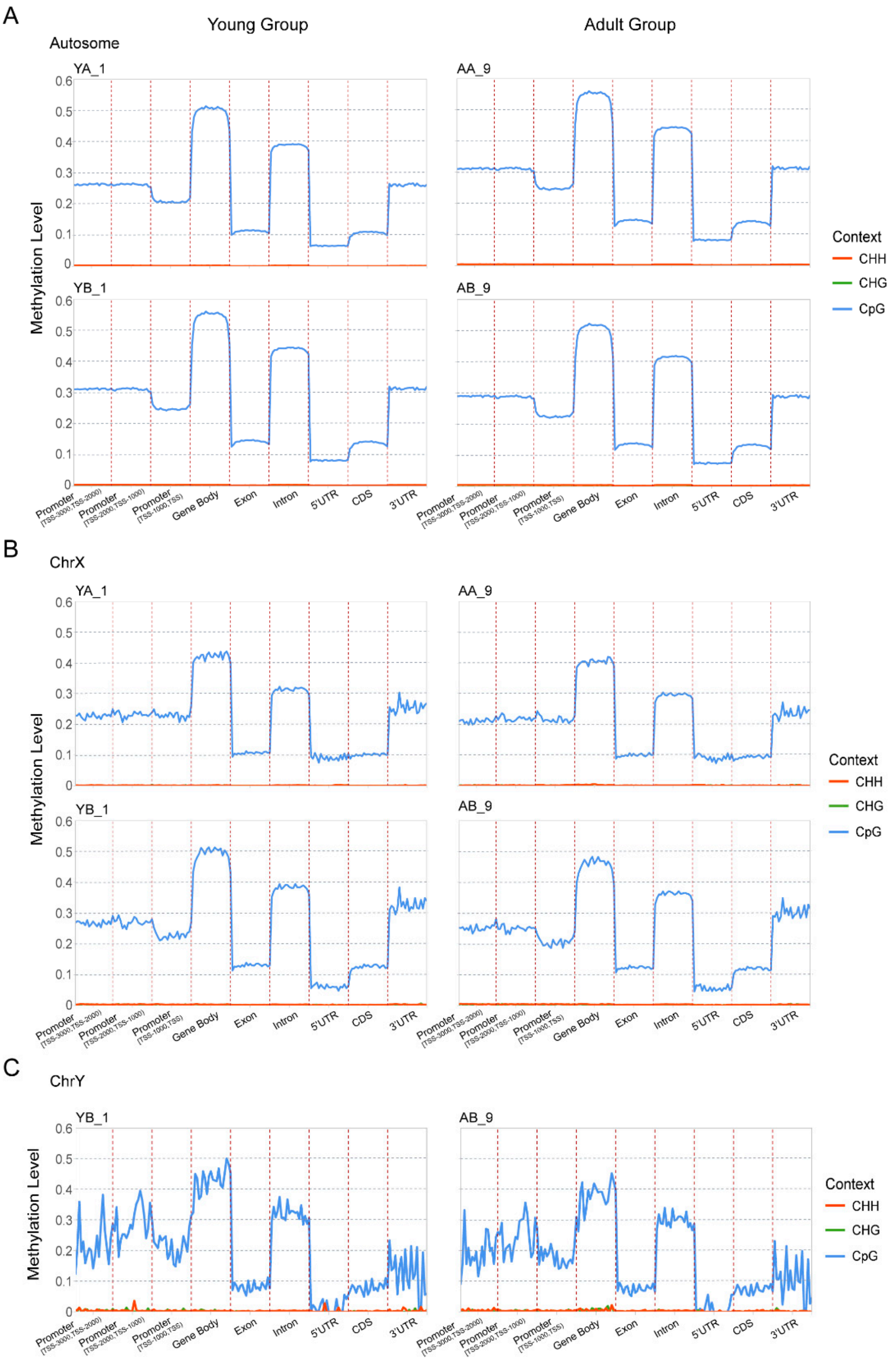

Figure S3. Methylation level on CHH, CHG, CpG contexts cytosine in different genomic annotations across the genome. (**A**) Distribution of methylation levels on different genomic elements on autosomes. (**B**) Distribution of methylation levels on different genomic elements on X chromosome. (**C**) Distribution of methylation levels on different genomic elements on Y chromosome. The x-axis represents different genomic elements, and the y-axis represents methylation level on CHH, CHG, CpG contexts cytosine. Each functional region of each gene is equally divided into 20 bins, and the methylation level of the corresponding bin in the functional region of all genes is averaged.

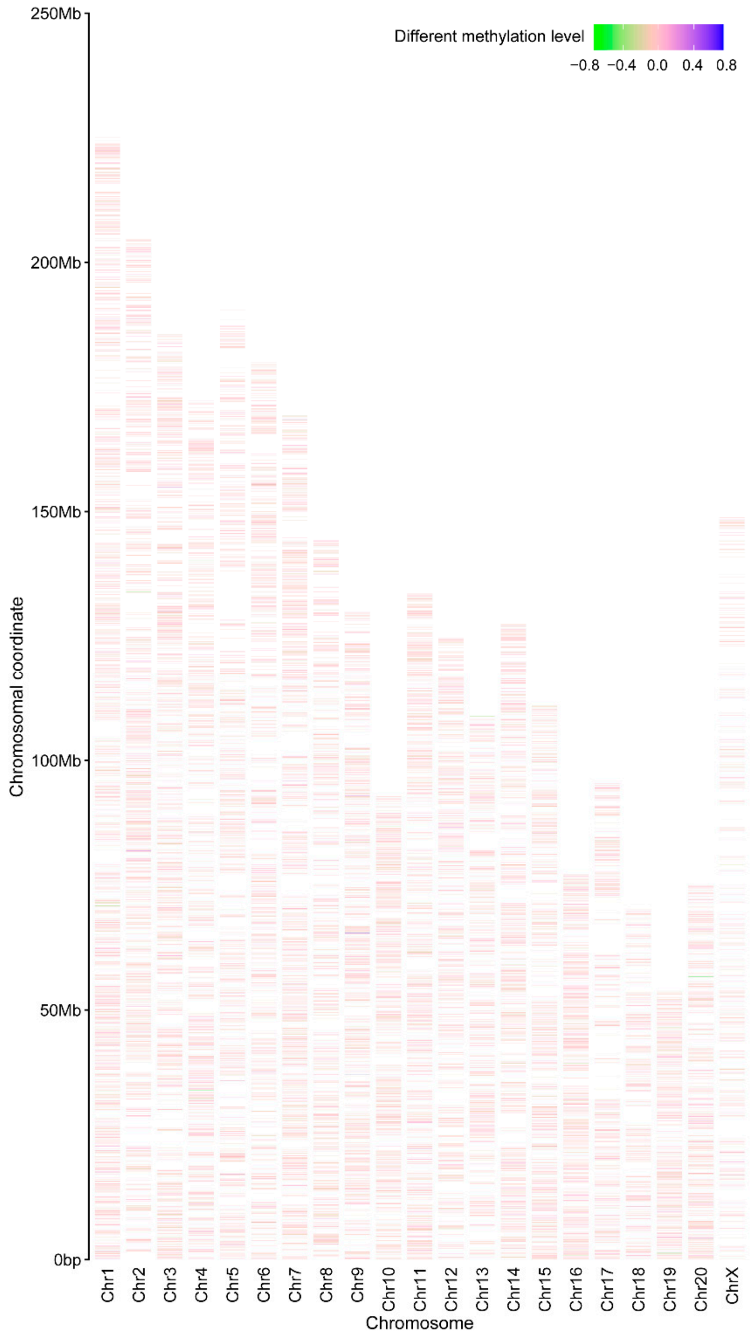

Figure S4. Distribution of different methylation regions (DMRs) on chromosomes

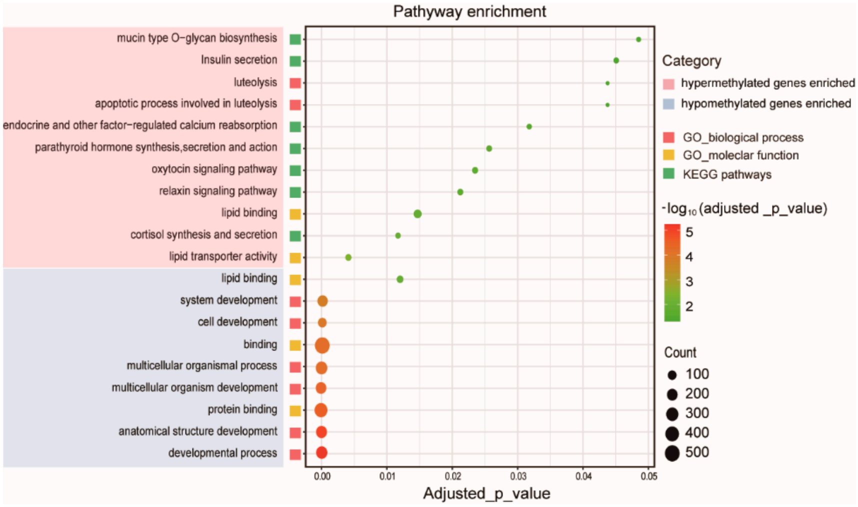

Figure S5. GO and KEGG pathway enrichment analysis of DMGs. Dot plot showing the up-regulated and down-regulated pathway terms (red, upregulated; blue, downregulated) by DMGs (FDR <0.05). PPI analysis of top 2,000 DMGs in adult group compared to young group.

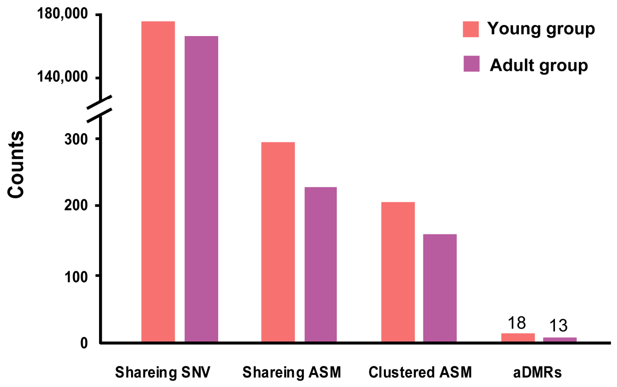

Figure S6. Statistics of shared SNV, shared ASM sites, cASM and aDMRs. Histogram depicting the number of shared SNV, shared ASM sites, cASM and aDMRs in young group and adult group.

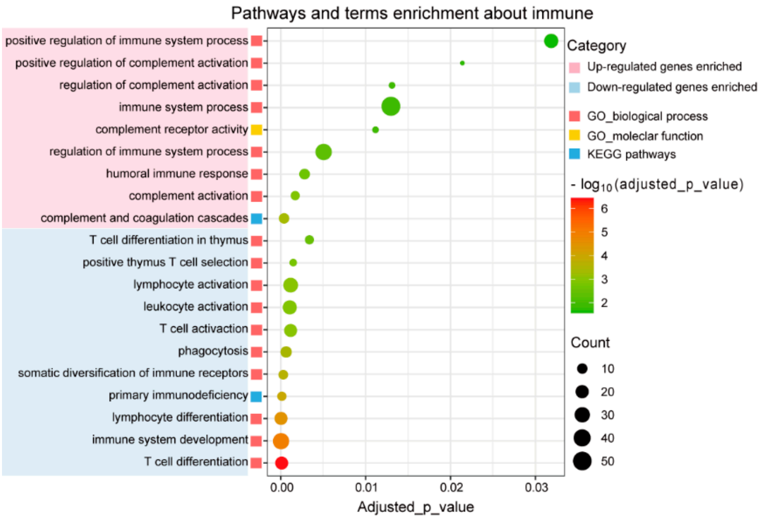

Figure S7. GO terms and KEGG pathways enrichment analysis of DEGs. Dot plot showing the up-regulated and down-regulated pathway terms about immune (red, upregulated; blue, downregulated) by DEGs (FDR <0.05).

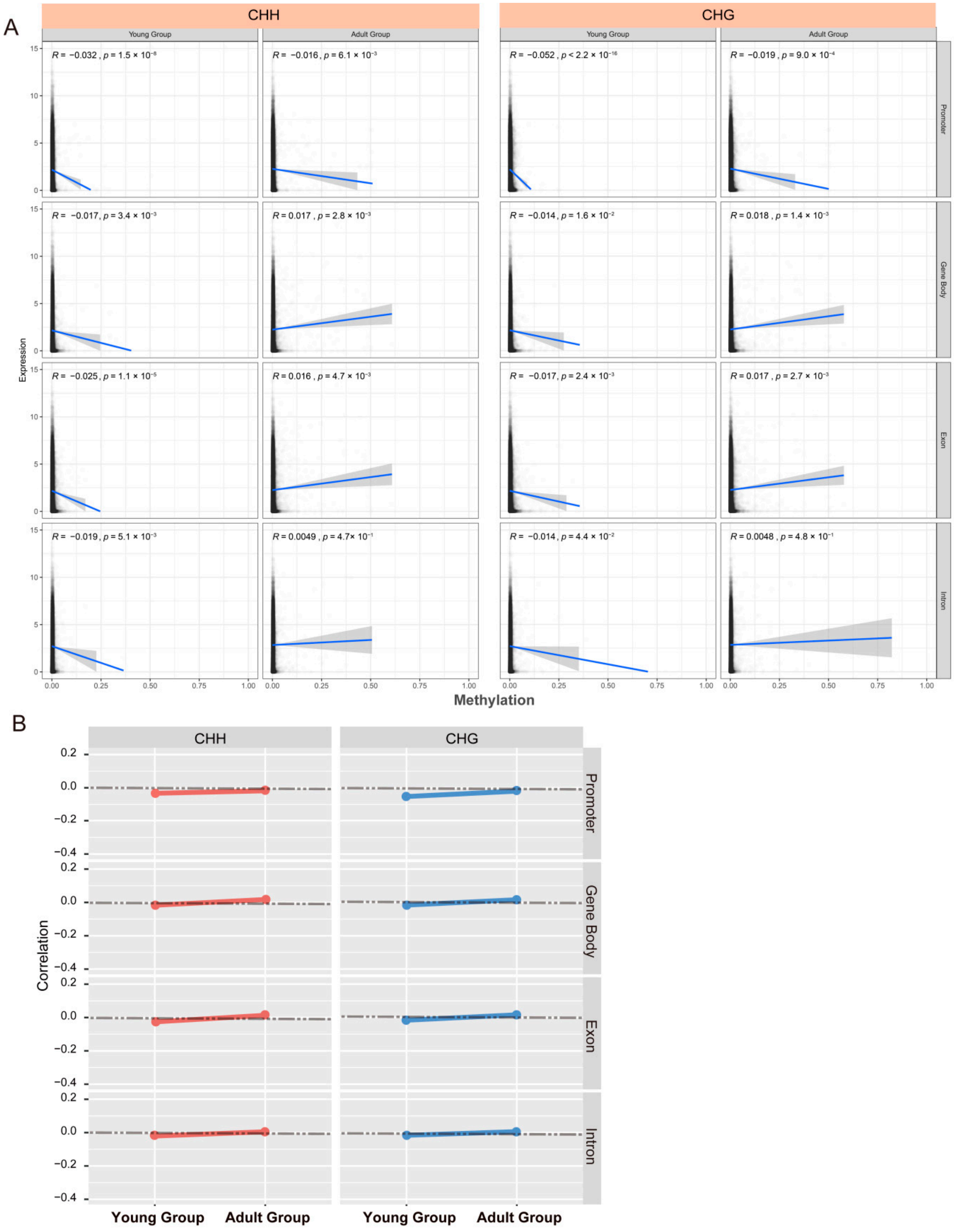

Figure S8. Relationship between methylation and expression. (**A**) Dot chart showing methtylation pairs stratified by age in columns and feature type in rows with 30,807 genes. Each dot represents the mean methylation level of CHHs and CHGs within the feature across all samples of that age on the x-axis and the log$_2$([mean TPM]+1) for the promoters,gene bodies, exons and introns on the y-axis. Linear regression with shaded standard error and the rho for each correlation is listed for each plot with Pearson method. (**B**) Correlation of feature expression and methylation stratified by cytosine context (columns) and feature type (rows).
